# In Their Words: African American and Latine Immigrant Older Adults (Re)Define Civic Participation

**DOI:** 10.1093/geroni/igaf122.423

**Published:** 2025-12-31

**Authors:** Laurent Reyes

**Affiliations:** University of California, Berkeley, Berkeley, California, United States

## Abstract

Older adults’ civic participation has received considerable attention. However, this literature has understudied the experiences of civic participation among minoritized ethnoracial older adults. Particularly absent from this literature is the contextualization of civic participation as it exists within cultural and historical structures of inequality that influence how these populations understand, participate, and experience civic life. A phenomenological design was used to explore civic participation through participants’ experiences and unique perspectives. Thirty-four in-depth, semistructured interviews were conducted with Latine immigrant and Black older adults (ages 60+) living in New Jersey and New York City. A conceptual content analysis was used to identify how older Black and Latine immigrant adults define civic participation for themselves. This study presents 3 new definitions of civic participation, that are derived directly from participants’ conceptualization and applied across the lived experiences. Definitions present civic participation as the responsibility of community belonging; as a religious/spiritual practice; and as a way of life. These definitions provide new perspectives by which to study civic participation and challenge current framing of helper and needy, altruism, the voluntary nature of participation, and the separation between social, political, and spiritual participation. Findings from this study contribute to expanding gerontology’s ontological imagination of how civic participation is experienced and conceptualized among older Latine immigrants and Black adults. The expertise shared by older African Americans and Latine immigrants lends us important perspectives to develop a critical theoretical framework by which scholars can more accurately study civic participation among this diverse population.

